# Supplementary material for: Novel Quercetin Derivative of 3,7-Dioleylquercetin Shows Less Toxicity and Highly Potent Tyrosinase Inhibition Activity
Source: Int J Mol Sci. 2021 Apr 20;22(8):4264. doi: 10.3390/ijms22084264 (PMC8072539; doi:10.3390/ijms22084264)
Supplement: Supplementary file 1 [file ijms-22-04264-s001.zip › ijms-1151681-supplementary.pdf]

Supplementary Materials

Novel Quercetin Derivative of 3,7-Dioleylquercetin Shows Less Toxicity and Highly Potent Tyrosinase Inhibition Activity

Moon-Hee Choi [1], Seung-Hwa Yang [1], Da-Song Kim [1], Nam Doo Kim [2], Hyun-Jae Shin [1]* and Kechun Liu [3]

[1] Department of Chemical Engineering, Graduate School of Chosun University, Gwangju 61452, Korea; aamoony1222@naver.com (M.-H.C.); sh556@daum.net (S.-H.Y.); dasong1214@daum.net (D.-S.K.); shinhj@chosun.ac.kr (H.-J.S.)

[2] VORONOI BIO Inc., Incheon 21984, Korea; namdoo@voronoibio.com

[3] Biology Institute, Qilu University of Technology (Shandong Academy of Sciences), Jinan 250103, Shandong Province, China; hliukch@sdas.org

* Correspondence: shinhj@chosun.ac.kr; Tel.: +82-62-230-7518

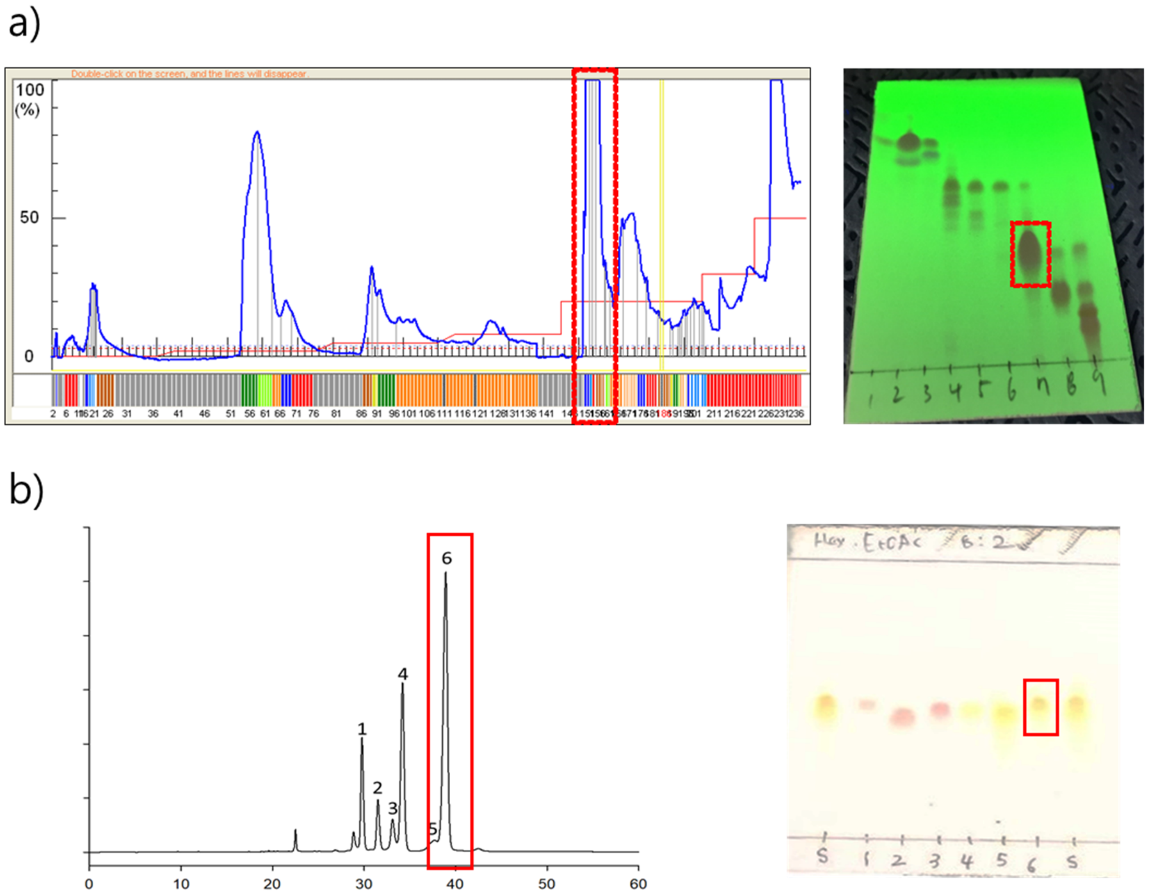

Figure S1. a) MPLC (Yamazen) and b) HPLC (Waters) separation, and TLC chromatograms of OQ synthesized and purified in this work. Plate: TLC silica gel 60 F254. Mobile phase: hexane/ethyl acetate mixture (8:2 v/v). OQ was visualized by *p*-anisaldehyde staining (*p*-anisaldehyde/sulfuric acid/ethanol mixture (0.5:0.5:9 v/v)). S, 1 2 3 4 5 6

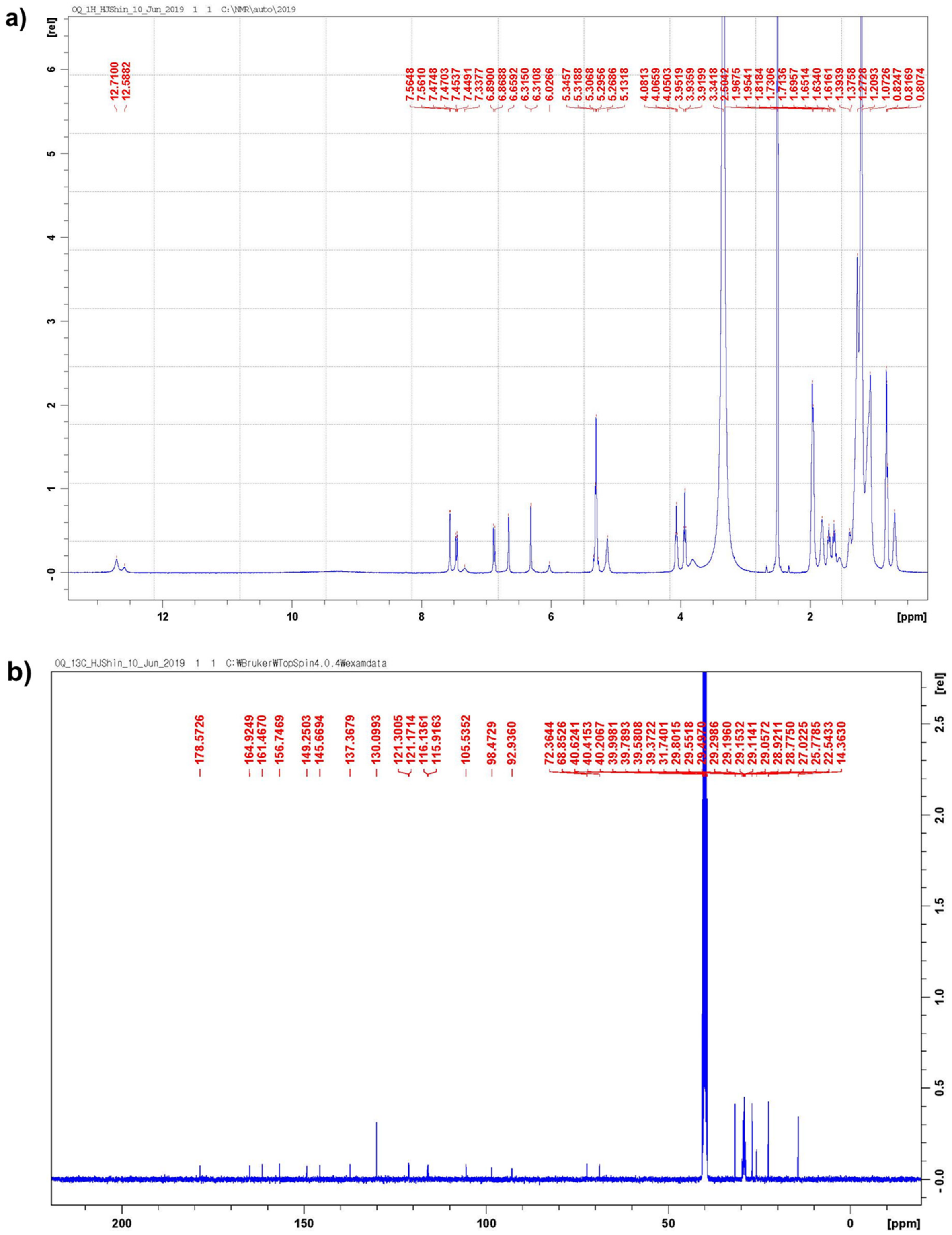
a)
OQ_1H_HJShin_10_Jun_2019 1 1 C:\NMR\auto\2019
12.7100
12.5882
7.5648
7.5610
7.4748
7.4703
7.4537
7.4491
7.3377
6.8900
6.8688
6.6592
6.3150
6.3108
6.0266
5.3457
5.3188
5.3068
5.2956
5.2686
5.1318
4.0813
4.0659
4.0503
3.9519
3.9359
3.9199
3.3418
2.5042
1.9675
1.9541
1.8184
1.7306
1.7136
1.6957
1.6514
1.6340
1.6161
1.3939
1.3758
1.2728
1.2093
1.0726
0.8247
0.8169
0.8074
[rel]
[ppm]
b)
OQ_13C_HJShin_10_Jun_2019 1 1 C:\Bruker\TopSpin4.0.4\examdata
178.5726
164.9249
161.4670
156.7469
149.2503
145.6694
137.3679
130.0993
121.3005
121.1714
116.1361
115.9163
105.5352
98.4729
92.9360
72.3644
68.8526
40.6241
40.4153
40.2067
39.9981
39.7893
39.5808
39.3722
31.7401
29.8015
29.5518
29.2986
29.1960
29.1532
29.1141
29.0572
28.9211
28.7750
27.0225
25.7785
22.5433
14.3630
[rel]
[ppm]

c)

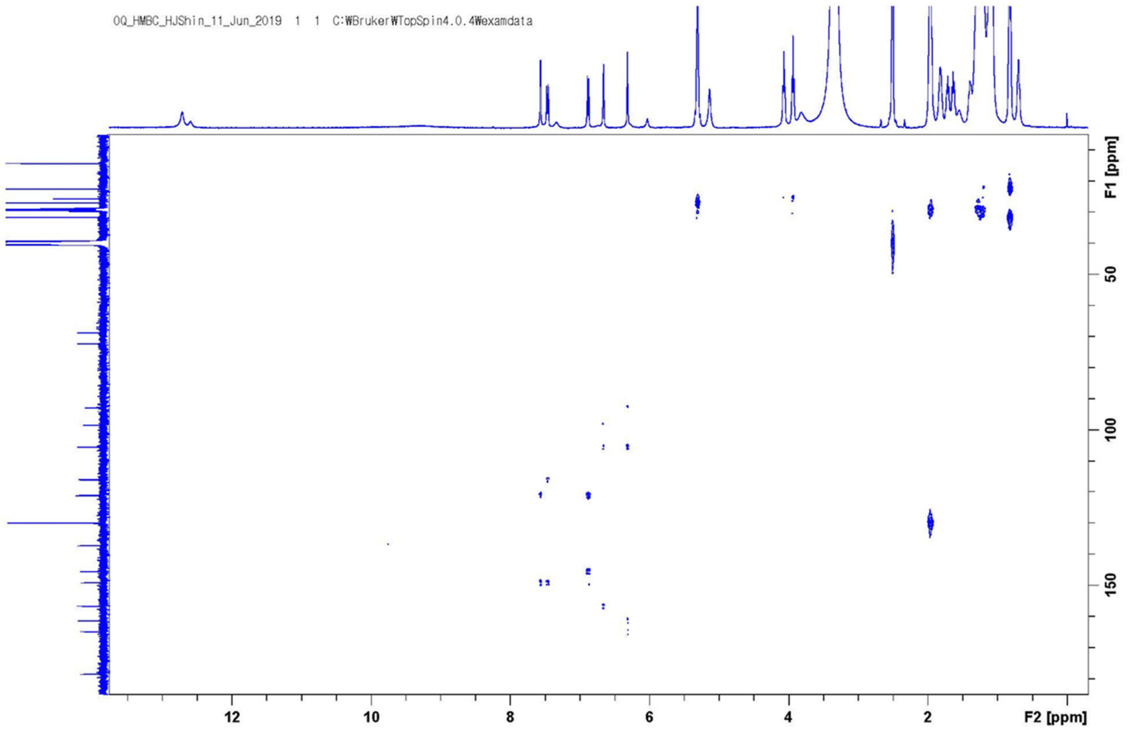
0Q_HMBC_HJShin_11_Jun_2019 1 1 C:\Bruker\TopSpin4.0.4\examdata
F1 [ppm]
50
100
150
12
10
8
6
4
2
F2 [ppm]

d)

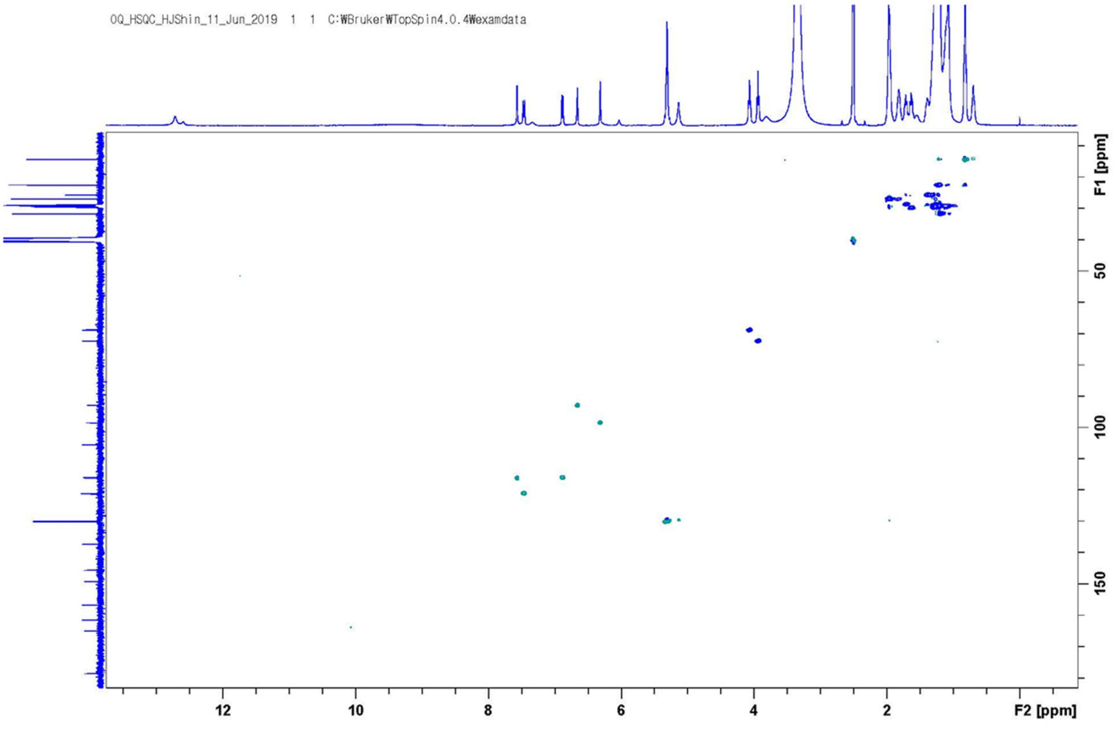
0Q_HSQC_HJShin_11_Jun_2019 1 1 C:\Bruker\TopSpin4.0.4\examdata
F1 [ppm]
50
100
150
12
10
8
6
4
2
F2 [ppm]

e)

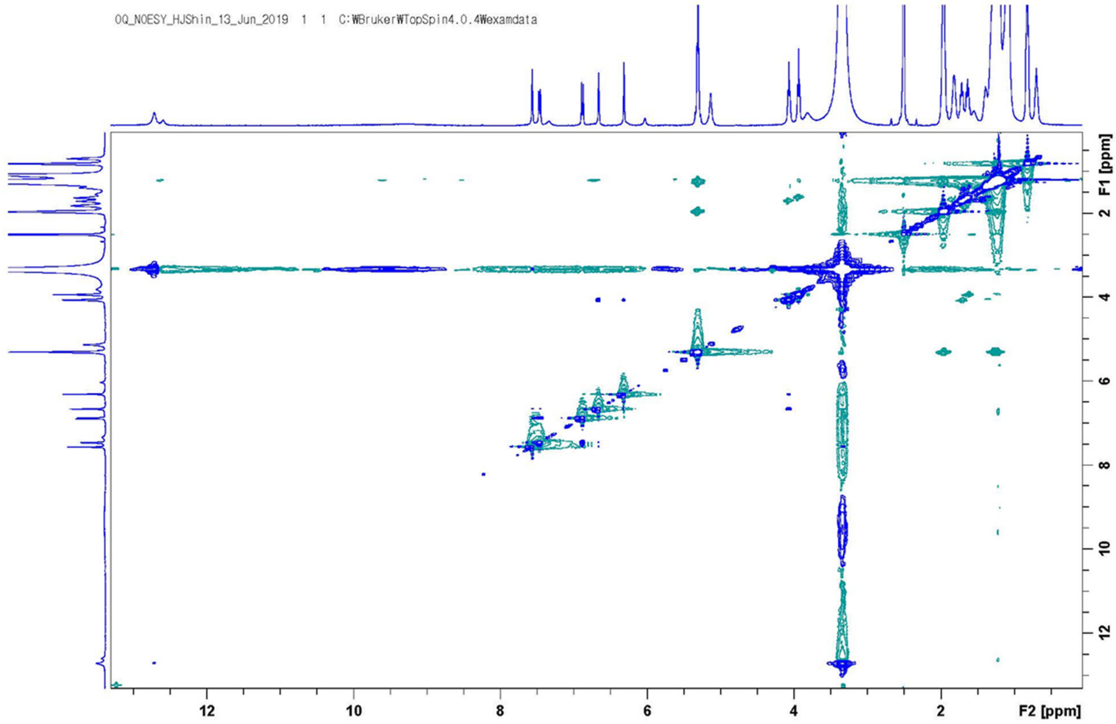

Figure S2. NMR spectroscopy of OQ synthesized and purified in this work. (a) ^{1}H-NMR spectroscopy, (b) ^{13}C-NMR spectroscopy, (c) 2D HMBC spectroscopy, (d) 2D HSQC spectroscopy, (e) 1H-1H NOESY spectroscopy. All peak assignments are described in the Materials and Methods.
